# Acute Effects of Whole-Body Vibration on the Postural Organization of Gait Initiation in Young Adults and Elderly: A Randomized Sham Intervention Study

**DOI:** 10.3389/fneur.2019.01023

**Published:** 2019-09-24

**Authors:** Arnaud Delafontaine, Thomas Vialleron, Matthieu Fischer, Guillaume Laffaye, Laurence Chèze, Romain Artico, François Genêt, Paul Christian Fourcade, Eric Yiou

**Affiliations:** ^1^CIAMS, Univ. Paris-Sud., Université Paris-Saclay, Orsay, France; ^2^CIAMS, Université d'Orléans, Orléans, France; ^3^ENKRE, Saint-Maurice, France; ^4^LBMC, Université de Lyon, Villeurbanne, France; ^5^UMR End:icap équipe 3, UFR des Sciences de la Santé Simone Veil, UVSQ, Montigny le Bretonneux, France

**Keywords:** whole-body vibration (WBV), gait initiation, elderly, young adults, anticipatory postural adjustment (APA)

## Abstract

Whole-body vibration (WBV) is a training method that exposes the entire body to mechanical oscillations while standing erect or seated on a vibrating platform. This method is nowadays commonly used by clinicians to improve specific motor outcomes in various sub-populations such as elderly and young healthy adults, either sedentary or well-trained. The present study investigated the effects of acute WBV application on the balance control mechanisms during gait initiation (GI) in young healthy adults and elderly. It was hypothesized that the balance control mechanisms at play during gait initiation may compensate each other in case one or several components are perturbed following acute WBV application, so that postural stability and/or motor performance can be maintained or even improved. It is further hypothesized that this capacity of adaptation is altered with aging. Main results showed that the effects of acute WBV application on the GI postural organization depended on the age of participants. Specifically, a positive effect was observed on dynamic stability in the young adults, while no effect was observed in the elderly. An increased stance leg stiffness was also observed in the young adults only. The positive effect of WBV on dynamic stability was ascribed to an increase in the mediolateral amplitude of “anticipatory postural adjustments” following WBV application, which did overcompensate the potentially destabilizing effect of the increased stance leg stiffness. In elderly, no such anticipatory (nor corrective) postural adaptation was required since acute WBV application did not elicit any change in the stance leg stiffness. These results suggest that WBV application may be effective in improving dynamic stability but at the condition that participants are able to develop adaptive changes in balance control mechanisms, as did the young adults. Globally, these findings are thus in agreement with the hypothesis that balance control mechanisms are interdependent within the postural system, i.e., they may compensate each other in case one component (here the leg stiffness) is perturbed.

## Introduction

Whole-body vibration (WBV) is a training method that exposes the entire body to mechanical oscillations while standing erect or seated on a vibrating platform. This method is nowadays commonly used by clinicians to improve specific motor outcomes in various sub-populations such as elderly and young healthy adults, either sedentary or well-trained. Specifically, it has been shown that WBV application might be beneficial to numerous lower limbs motor outcomes, such as endurance, power, strength, neuromuscular activity, flexibility, and knee stability [for young healthy subjects, see for example (1–7); for elderly, see for example (8–13), for review see (14)].

Balance and locomotion might also be improved by WBV application, as quantified (i) with center of pressure (COP) measures in static upright condition [for young healthy adults, see for example (3, 15, 16); for elderly, see for example (17–20)], and (ii) with commonly-used clinical tests such as the single-leg-stance test, the Tinetti test, the Timed-Up-and-Go test, the Berg Balance Scale, and the Limits of stability. However, it is well-recognized that these classical clinical tests are subjective, show ceiling effects, and are usually not responsive enough to measure small progresses or deteriorations in a subject's ability to balance (21, 22). Mancini and Horak (22) further stressed that the greatest limitation of this clinical approach to rating balance is that it cannot specify what type of balance problem a subject suffers in order to direct a treatment. Upright balance maintenance during locomotor tasks is indeed a highly complex task since it necessitates the coordination between many complementary postural mechanisms, including dynamic postural phenomena before the swing foot clearance (23–25), the regulation of stance leg stiffness (26), the swing foot placement on the support surface (27), the braking of center of mass fall (28–30) etc. The investigation of these mechanisms [cf. description below; see also (31) for a recent review] requires an in-depth biomechanical analysis with force plate recordings. Such analysis is not provided in the classical clinical tests reported above. In fact, the classical clinical test, as Timed Up and Go test for example, could be limited to determined individual risk of falls in elderly (32). Furthermore, Topper et al. (33) show that falls happened most frequently in elderly subjects scoring poorly on clinical tests emphasizing transfer of quasi-static to dynamic situations.

Gait initiation is a functional task that is classically used in the literature as an experimental paradigm to investigate how the central nervous system (CNS) controls balance in dynamical conditions in both healthy adults [e.g., (31–35) for recent articles] and pathological patients [e.g., (36–40) for recent articles]. Gait initiation appears to be the major biomechanical model, complementary to the classical clinical test, in order to predict postural disorders in elderly fallers and non-fallers (41–44). Therefore, it seems highly appropriate for an investigation on the effects of WBV on dynamic balance. Gait initiation corresponds to the transient period between static erect posture and steady state walking (23–25). It can be divided into two successive phases: a postural phase that precedes the onset of the swing heel off (the so called “anticipatory postural adjustments”, APA) followed by an execution phase that ends at the time of swing foot contact (FC) with the ground.

During APA, the COP is shifted backward, creating the initial forward propulsive forces that are necessary to reach the intended center of mass (COM) velocity and step length (23, 25, 45). At the same time, the COP is shifted laterally toward the swing leg, which acts to propel the COM toward the stance leg (23, 26, 35, 46, 47). This anticipatory mediolateral postural dynamic acts to attenuate the COM fall toward the swing leg during the execution phase under gravity (26, 48, 49). It has therefore a stabilizing function. In case mediolateral APA are not sufficient to ensure stabilization, a strategy of lateral swing foot placement that enlarges the base of support size has been documented (27, 46, 47, 50, 51). The development of APA and the lateral swing foot placement during locomotor tasks are known to be dependent on somatosensory inputs from the lower limbs (52–55). The perturbation of these inputs by the applications of WBV might thus alter these two mechanisms and increase instability and/or reduce motor performance.

In addition to mediolateral APA and lateral swing foot placement, recent modeling study showed that increasing the stance leg stiffness along the mediolateral direction would result in a larger COM fall toward the swing leg side, thus leading to an increased mediolateral instability. The application of WBV may trigger a “tonic vibration reflex” (TVR) via the activation of muscle spindles of the lower limbs (56, 57). Although controversial (8, 58–60), this TVR might potentially be responsible for an increase in lower limbs stiffness and may thus have a negative impact on stability.

Globally, this brief literature review suggests that WBV may potentially perturb the balance control mechanisms involved in gait initiation and may therefore induce instability. This statement thus markedly contrasts with the results of the above-reported clinical tests, which rather suggest that WBV may be effective in improving relatively basic balance ability and locomotion. Thus, the purpose of this study is to clarify the effects of acute WBV application on the balance control mechanisms during gait initiation in young healthy adults and elderly. It is hypothesized that the balance control mechanisms at play during gait initiation may compensate each other in case one or several components are perturbed following acute WBV application, so that postural stability and/or motor performance can be maintained (or even improved). It is further hypothesized that this capacity of adaptation is altered with aging, with a consequent increased instability following WBV application.

## Methods

### Participants

The study is a randomized investigation that includes 81 healthy adults (i.e., 41 young and 40 elderly). The non-probability convenience method is used, i.e., participants were randomly assigned to one of the four following treatment groups using the “envelope method” (see below): (1) 20 young adults (11 men and 9 women, mean age 25.3 ± 3.5 years, height 1.71 ± 0.05 m and body-mass 71.4 ± 5.3 kg) were assigned to the Young WBV training group (YWBV); (2) 21 matched young adults were assigned to the Young Sham Group (YSG) (11 men and 10 women, mean age 26.6 ± 4.2 years, height 1.74 ± 0.04 m and body-mass 72.1 ± 4.9 kg); (3) 20 elderly (9 men and 11 women, mean age 83.5 ± 2.8 years, height 1.69 ± 0.06 m and body-mass 70.4 ± 4.3 kg) were assigned to the Elderly WBV training group (EWBV) and (4) 20 matched elderly (9 men and 11 women, mean age 84.2 ± 3.7 years, height 1.71 ± 0.07 m and body-mass 71.7 ± 3.9 kg) were assigned to the Elderly Sham Group (ESG; see Table 1). In the “envelope method,” each participant chooses a closed envelope in which his/her treatment group is indicated in a code form. The participant does not have access to this code but the experimenters do (single blinded condition).

**Table 1:** Anthropometric data of subjects in both WBV and sham groups (young adults and elderly).

**Group**	**Variables**	**WBV group**	**Sham group**	***P*-values**
Young adults (*n* = 41)	Women (*n*)	9	10	NS
	Men (*n*)	11	11	NS
	Age (years)	25.30 ± 3.50	26.6 ± 4.2	NS
	Height (m)	1.71 ± 0.05	1.74 ± 0.04	NS
	Body mass (kg)	71.40 ± 5.30	72.10 ± 4.90	NS
Elderly (*n* = 40)	Women (*n*)	11	11	NS
	Men (*n*)	9	9	NS
	Age (years)	83.50 ± 2.80	84.20 ± 3.70	NS
	Height (m)	1.69 ± 0.06	1.71 ± 0.07	NS
	Body mass (kg)	70.40 ± 4.30	71.70 ± 3.90	NS

*Reported values are means ± standard deviation*.

*NS, non-significant difference*.

The two WBV groups (young adults and elderly) received a single WBV training and the two sham groups received a placebo treatment (cf. below for description). As stated above, participants were blinded to their allocation group. Participants had no recent history of trauma, known metabolic disorders, inflammatory infectious arthropathies, bone malignancies or neurological disease. In addition, participants were all naïve about WBV treatment. They all gave written consent after having been informed of the nature and purpose of the experiment which was approved by local ethics committees from the CIAMS Research Unit, Equipe d'Accueil (EA) 4532. The study complied with the standards established by the Declaration of Helsinki and was assigned the following trial registration number: 2017-002539-40.

### Experimental Task and Conditions

Experiments took place in the Biomechanics laboratory of the Paris-Saclay University located within the Kremlin-Bicêtre Hospital (Paris, France). Physical conditions (room temperature and time of the day) were common to all treatment groups, and the same before/after the WBV treatment.

Participants initially stood barefoot in a natural upright posture on a force plate (0.9 × 1.80 m, AMTI, Watertown, USA) embedded at the beginning of a 6 m walkway track. The feet were shoulder-width apart, with the arms alongside the trunk and the gaze directed forward to a small target at eye level (2 cm diameter, 5 m distant). The locations of the heel and big toe of each foot in the initial posture were marked with sections of adhesive tape placed on the force plate and were used as a visual reference on which participants positioned themselves under the supervision of the experimenters. From this initial posture, participants performed two series of 10 gait initiation trials: one series just before, and a second series immediately after a specific treatment (pre- and post-treatment conditions, respectively) depending on their group (WBV or sham). In both pre- and post-treatment conditions, participants initiated gait at a spontaneous velocity and at their own initiative following an auditory signal delivered by the experimenter, and then continued walking straight until the end of the track. Participants initiated gait with their preferred leg in all trials. One blank trial was provided in the pre-manipulation condition (not recorded) to ensure that the instructions were well-understood by the participant and that the material was operational. The rest time was ~10 s between trials.

### WBV and Placebo Treatment

For both the WBV group and the sham group, participants stood upright on a vibration platform (referee Power Fitness Double D®) with knees flexed ~30° and barefoot. During the treatment, knee angle was monitored on-line by both the subject him/herself and the experimenters thanks to a visual feedback provided by the signal of a monoaxial electrogoniometer (Biometrics®) fixed on the right leg. Participants were instructed to maintain the signal within a 25–35 range degrees.

For both young adults and elderly, the WBV application included four 45 s bouts of vibration, intercalated by 1 min of seated rest without vibration between bouts (61–63). Vibrations were delivered by the vibration platform at a frequency of 50 Hz [this frequency is known to enhance locomotor task and intralimb coordination (61)] with a vertical displacement of 2 mm and a 2 g acceleration.

The sham group received a treatment that followed exactly the same procedure as the WBV group except that no “real” vibrations were applied to participants. In order to maintain the most realistic placebo conditions as possible, participants were instructed by an experimenter that they could not physically feel the vibrations because they were too small to be sensed.

### Mechanical Model

The same mechanical model as in a previous study was used to determine the stance leg stiffness along the mediolateral direction during the execution phase of gait initiation [for details on this model and the related equations of motion, see (26)]. In brief, the human body was modeled as a single conic inverted pendulum which rotates around a fixed point corresponding to the stance ankle. It was considered that the COM falls laterally under the influence of two forces: the gravity force P = mg (where m is the mass of the whole body, and g is the gravitational acceleration) and an elastic restoring mediolateral force T (in Newtons) that reflects active muscular control of the movement (64, 65), with T = k|yM| where k is the stance leg stiffness (Newtons/meter) and |yM| is the absolute value of the mediolateral COM shift (in meters), systematically oriented toward the swing leg (positive values) during the execution phase. The initial position and velocity of the cone corresponded to the position and velocity of the participant's COM at toe off.

### Raw Data

Force-plate data were low-pass filtered using a second order Butterworth filter with a 10 Hz (30) cut-off frequency. The mediolateral (yp) and anteroposterior (xp) coordinates of the COP (in meters) were computed from force-plate data as follows:

(1) $$\begin{array}{l} {\text{y}}_{\text{p}}=\frac{\text{Mx}+\text{Fy}\times \text{dz}}{\text{Fz}} \end{array}$$

(2) $$\begin{array}{l} {\text{x}}_{\text{p}}=\frac{-\text{My}+\text{Fx}\times \text{dz}}{\text{Fz}} \end{array}$$

where Mx and My are the moments (in Newtons.meters) around the anteroposterior and mediolateral axes (at the force-plate origin), respectively; Fx, Fy, and Fz (in Newtons) are the mediolateral, anteroposterior, and vertical ground reaction forces, respectively; and dz (in meters) is the distance between the surface of the force-plate and its origin which is located below this surface.

Instantaneous COM acceleration along the anteroposterior and mediolateral axes was determined from the ground reaction forces according to the Newton's second law. COM velocity and displacement were computed by successive numerical integrations of COM acceleration using the rectangles method (66) and integration constants equal to zero, i.e., initial velocity and displacement null (23).

### Experimental Dependent Variables

The biomechanical traces and the representations of the main experimental variables obtained for one representative young subject of the WBV group initiating gait (one trial) in the pre-treatment condition and post-treatment condition are presented in the Figure 2. The following instants were determined from the biomechanical traces: gait initiation onset (t_0_), swing heel-off, swing toe-off, swing foot-contact, and rear foot-off. These instants were determined from force-plate data (23, 67, 68). Two t_0_ times were estimated, one for the mediolateral axis and one for the anteroposterior axis. The t_0_ times corresponded to the instants when the mediolateral or anteroposterior COP trace deviated 2.5 standard deviations from its baseline value.

The mediolateral and anteroposterior COM position in the initial upright static posture were estimated by averaging the COP position during the 250 ms period preceding the “all set” signal. Gait initiation was divided into APA (from t_0_ to heel-off), swing foot-lift (from heel-off to toe-off), and execution phase (from toe-off to foot-contact). The duration of APA along the mediolateral and anteroposterior axes were computed separately, because the t_0_ times for these two axes do not necessarily occur simultaneously. The amplitude of APA was characterized by the peaks of the backward and mediolateral COP shift obtained during the APA time window. COM velocity and displacement along the mediolateral and anteroposterior axes were quantified at heel-off and foot-contact.

The spatiotemporal features of gait performance during the execution phase included: the swing phase duration, the anteroposterior COM velocity at foot-contact (progression velocity), and the step length. Step length corresponds to the difference between the most backward COP position during gait initiation and the COP position at the time of rear foot-off (69). Rear foot off time was marked with the mediolateral COP trace.

The spatiotemporal features of postural stability during the execution phase included: the step width, the ML COM position at FC, the ML COM velocity at FC and the margin of stability (MOS). An adaptation of the MOS introduced by Hof et al. (70) was used to quantify the mediolateral dynamic stability at foot-contact (thereafter referred to as “stability”). The MOS corresponds to the difference between the mediolateral boundary of the base of support (BOSymax, in meters) and the mediolateral position of the “extrapolated COM” at swing foot-contact (_y_COMFC, in meters). Thus:

(3) $$\begin{array}{l} \text{MOS}=\text{BOSymax}{-}_{\text{y}}\text{COMFC} \end{array}$$

Participants systematically landed on the force-plate with the swing heel first, then the toe. Under such foot landing strategy, it is known that BOSymax could be estimated with the mediolateral COP position at the time of rear foot-off (69, 71). Based on the study by Hof et al. (70) and the results from our previous studies (26, 27, 69, 71, 72) the mediolateral position of the extrapolated COM at foot-contact (_y_COMFC) was calculated as follows:

(4) CyOMFC = yMFC+y′MFCω0

where yM_FC_ and y'M_FC_ are respectively the mediolateral COM position (in meters) and velocity at foot-contact (in meters/second), and ω_0_ is the eigenfrequency of the body (Hz), modeled as an inverted pendulum and calculated as follows:

(5) $$\begin{array}{l} \omega_{0}=\sqrt{\frac{g}{l}} \end{array}$$

where g = 9.81 m/s^2^ is the gravitational acceleration and *l* is the length of the inverted pendulum, which in this study corresponded to 57.5% of body height (73). Mediolateral dynamic stability at foot-contact is preserved on the condition that _y_COMFC is within BOSymax, which corresponds to a positive MOS. A negative MOS indicates a mediolateral instability and implies that a corrective action is required to maintain balance. Hence, variables related to the mediolateral stability included the MOS and its components, i.e., step width, mediolateral COM velocity and position at swing foot-contact. Step width corresponds to the difference between the most lateral position of the mediolateral COP trace obtained during the first plateau of the trace and the mediolateral COP position at the time of rear foot-off (69).

### Statistics

Mean values and standard deviations were calculated for each variable in each condition. The normality of data was checked using the Kolmogorov–Smirnov test and the homogeneity of variances was checked using the Bartlett test. Repeated measures (RM) ANOVAs with the treatment (pre-treatment condition vs. post-treatment condition) as within subject factor and the group (sham group vs. WBV group) as between subjects' factor were used on each experimental variable. Because this research focuses on the effect of WBV application on gait initiation in young and elderly adults, merely than on the effects of age on gait initiation, the RM ANOVAs were conducted for young and elderly adults taken separately. When necessary, a significant outcome was followed-up with the Tukey *post-hoc* test. Finally, linear correlation between variables was assessed with the Pearson coefficient *r*. The threshold of significance was set at *p* < 0.05.

## Results

### Biomechanical Traces

**Figure 1:**
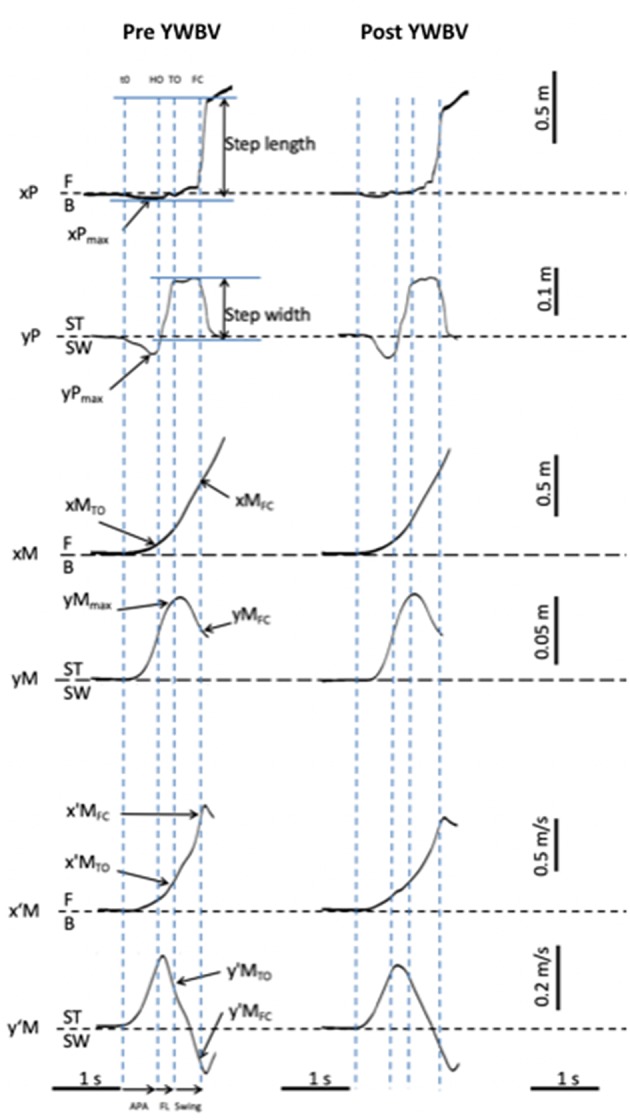
Typical biomechanical traces and representation of the main experimental variables obtained in one young adult of the WBV group initiating gait in the pre and post treatment condition. Anteroposterior (AP) direction (x axis): x′M: center of mass (COM) velocity; x′M_TO_ and x′M_FC_: COM velocity at toe off and foot contact. xM: COM displacement; xM_TO_ and xM_FC_: COM displacement at toe off and foot contact; xP: center of pressure (COP) displacement; xP_max_: peak of COP displacement during the anticipatory postural adjustments; F: forward; B: backward. Mediolateral (ML) direction (x axis): y′M: COM velocity; y′M_TO_ and y′M_FC_: COM velocity at toe off and foot contact; yM: COM displacement; yM_TO_ and yM_FC_: COM displacement at toe off and foot contact; yP: COP displacement; yP_max_: peak of COP displacement during the APA; ST: stance limb; SW: swing limb. Vertical dashed lines t0: onset variation of biomechanical traces; HO: swing heel off; TO: swing toe off; FC: swing foot contact. Horizontal arrows: APA: anticipatory postural adjustments phase; FL: foot lift phase; swing: execution phase.

The time course of the biomechanical traces was globally similar in the two groups (young adults and elderly) and in the different treatment conditions (WBV and sham). The traces obtained in a representative young adult subject, before and after the WBV treatment, are reported in the Figure 1.

**Figure 2:**
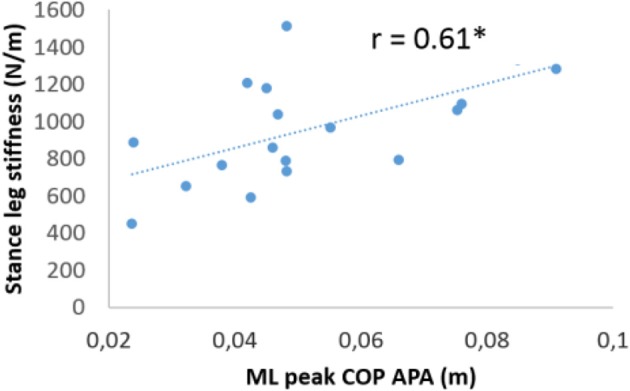
Linear regression between normalized medio-lateral peak anticipatory center-of-pressure (COP) shift and the normalized medio-lateral stance leg stiffness in the WBV condition. Each point represents the average value of subjects in the WBV condition (*r* = 0.61, *p* < 0.01).

As can be seen from this figure, swing heel off was systematically preceded by postural dynamics that corresponded to APA. During these APA, the COP displacement reached a peak value in a backward direction (see the negative variation of the xP trace) and toward the swing leg side (negative variation of the yP trace), while the COM velocity was directed forwards (positive variation of the x'M trace) and toward the stance leg side (positive variation of the y'M trace). The mediolateral COM velocity trace reached a first peak value toward the stance leg side at around heel off. This trace, then fell toward the swing leg side and a second peak value toward this side was reached a few milliseconds after foot contact. The anteroposterior COM velocity increased progressively until it reached a peak value a few milliseconds after swing foot contact. The mediolateral COM shift trace was bell-shaped and reached a peak value toward the stance leg side at the beginning of the swing phase, while the anteroposterior COM was continuously shifted forward (see Figure 2). Differences across the conditions are reported in the paragraphs below.

### Initial Posture, Anticipatory Postural Adjustments, and Foot-Lift Phase

In both the young adults and the elderly, no baseline differences were found between the WBV and sham groups (*p* > 0.05) for all the variables (see Table 2).

**Table 2:** Comparison of APA and foot lift parameters between the pre- and post-treatment condition in the WBV and sham group (young adults and elderly).

	**APA and Foot lift phase**	**WBV group**		**Sham group**		**Group X treatment interaction**
	**Variables**	**Pre-treatment**	**Post-treatment**	**Pre-treatment**	**Post-treatment**	***P*-values**
Young adults	AP COP shift (cm)	4.94 ± 1.51	5.29 ± 1.23	5.44 ± 1.37	5.52 ± 1.41	NS
	ML COP shift(cm)	4.61 ± 2.17	5.35 ± 2.09^***^	4.99 ± 1.27	5.12 ± 1.43	<0.001
	AP APA duration (s)	0.05 ± 0.11	0.47 ± 0.01	0.51 ± 0.10	0.50 ± 0.09	NS
	ML APA duration (s)	0.45 ± 0.08	0.47 ± 0.09	0.49 ± 0.01	0.45 ± 0.07	NS
	Foot lift duration (s)	0.12 ± 0.02	0.12 ± 0.03	0.14 ± 0.04	0.13 ± 0.04	NS
	AP COM velocity at HO (m/s)	0.21 ± 0.04	0.22 ± 0.05	0.23 ± 0.04	0.22 ± 0.04	NS
	ML COM velocity at HO (m/s)	0.14 ± 0.04	0.15 ± 0.04^**^	0.15 ± 0.02	0.15 ± 0.03	0.04
Elderly	AP COP shift (cm)	4.53 ± 1.23	5.03 ± 1.32	4.69 ± 0.64	4.95 ± 1.27	NS
	ML COP shift(cm)	4.21 ± 1.55	4.48 ± 1.63	4.85 ± 1.53	5.05 ± 1.88	NS
	AP APA duration (s)	0.40 ± 0.09	0.36 ± 0.06	0.38 ± 0.06	0.36 ± 0.05	NS
	ML APA duration (s)	0.39 ± 0.09	0.37 ± 0.05	0.36 ± 0.05	0.37 ± 0.05	NS
	Foot lift duration (s)	0.14 ± 0.02	0.14 ± 0.02	0.14 ± 0.02	0.14 ± 0.04	NS
	AP COM velocity at HO (m/s)	0.15 ± 0.05	0.16 ± 0.05	0.14 ± 0.03	0.15 ± 0.05	NS
	ML COM velocity at HO (m/s)	0.11 ± 0.04	0.11 ± 0.03	0.12 ± 0.04	0.12 ± 0.04	NS

*Reported values are means ± standard deviation. APA, anticipatory postural adjustments; COM, center of mass; COP, center of pressure; AP, anteroposterior; ML, mediolateral; HO, heel off; NS, non-significant interaction*.

*, **, *** *Significant difference between the pre and post-treatment condition as revealed with the Tukey post-hoc test, with P < 0.05, P < 0.01, and P <0.001, respectively*.

In both the young adults and the elderly, statistical analysis showed that there was no significant effect of the group (sham group vs. WBV group) or treatment (pre-treatment condition vs. post-treatment condition) nor any significant interaction between these two factors on the initial COM position along the mediolateral or anteroposterior direction.

In the elderly, there was also no significant effect of the group or treatment nor any significant interaction between these two factors on the spatiotemporal parameters of APA along the mediolateral or anteroposterior direction (APA duration, peak COP shift, COM shift, and velocity at heel-off), nor on the swing foot-lift phase duration. In contrast, in the young adults, there was a significant effect of the treatment [*F*_(1, 39)_ = 17.94, *p* < 0.001] on the mediolateral peak of anticipatory COP shift, along with a significant group X treatment interaction [*F*_(1, 39)_ = 8.83, *p* < 0.01]. Similarly, there was a significant effect of the treatment [*F*_(1, 39)_ = 8.89, *p* < 0.01], along with a significant group X treatment interaction [*F*_(1, 39)_ = 4.38, *p* = 0.04] on the mediolateral COM velocity at heel-off. *Post-hoc* analysis further revealed that these two interactions could be ascribed to the result that none of these two APA related-parameters were significantly different between the pre and post-treatment condition for the sham group, while both were significantly larger in the post-treatment condition than in the pre-treatment condition for the WBV group (*p* < 0.001 for the mediolateral peak of anticipatory COP shift and *p* < 0.05 for the mediolateral COM velocity at heel-off; cf. Table 2).

### Motor Performance

In both the young adults and the elderly, no baseline differences were found between the WBV and sham groups (*p* > 0.05) for all the variables (see Table 3).

**Table 3:** Comparison of motor performance, stability and stiffness parameters between the pre- and post-treatment condition in the WBV and sham group (young adults and elderly).

	**Motor performance, stability and stiffness**	**WBV group**		**Sham group**		**Group X treatment interaction**
	**Variables**	**Before**	**After**	**Before**	**After**	***P*-values**
Young adults	Swing phase duration (s)	0.12 ± 0.02	0.13 ± 0.03	0.14 ± 0.04	0.14 ± 0.04	NS
	Step length (cm)	67.94 ± 6.43	67.89 ± 6.74	70.32 ± 8.61	70.68 ± 7.45	NS
	AP COM velocity at FC (m/s)	0.96 ± 0.01	0.98 ± 0.09	1.01 ± 0.12	1.02 ± 0.11	NS
	Step width (cm)	17.11 ± 4.03	17.07 ± 4.09	18.12 ± 4.57	17.52 ± 4.19	NS
	ML COM position at FC (cm)	3.56 ± 2.80	4.52 ± 2.11^**^	3.85 ± 2.27	3.93 ± 2.31	0.03
	ML COM velocity at FC (m/s)	−0.14 ± 0.05	−0.12 ± 0.04^**^	−0.15 ± 0.03	−0.14 ± 0.03	0.04
	Margin of stability (cm)	4.74 ± 3.43	5.76 ± 2.56^*^	5.79 ± 2.65	5.77 ± 2.48	0.02
	Stiffness	835.10 ± 355.40	975.62 ± 290.11^*^	973.32 ± 293.83	972.98 ± 328.87	0.04
Elderly	Swing phase duration (s)	0.14 ± 0.02	0.14 ± 0.04	0.14 ± 0.02	0.14 ± 0.04	NS
	Step length (cm)	52.74 ± 10.19	53.89 ± 8.35	52.74 ± 10.32	54.39 ± 9.20	NS
	AP COM velocity at FC (m/s)	0.74 ± 0.17	0.78 ± 0.11	0.76 ± 0.14	0.79 ± 0.11	NS
	Step width (cm)	16.84 ± 2.44	17.17 ± 2.77	17.49 ± 4.42	18.10 ± 4.96	NS
	ML COM position at FC (cm)	6.32 ± 5.19	5.98 ± 4.84	6.38 ± 6.34	6.11 ± 5.66	NS
	ML COM velocity at HC (m/s)	−0.13 ± 0.03	−0.14 ± 0.03	−0.14 ± 0.04	−0.15 ± 0.05	NS
	Margin of stability (cm)	5.98 ± 1.72	5.18 ± 2.21	6.38 ± 2.24	5.59 ± 2.58	NS
	Stiffness	1061.90 ± 145.99	1006.45 ± 221.21	1208.88 ± 389.14	1208.33 ± 508.51	NS

*Reported values are means ± standard deviation. COM, center of mass; COP, center of pressure; AP, anteroposterior; ML, mediolateral; FC, foot contact; NS, non-significant interaction*.

*, **, *** *Significant difference between the pre and post-treatment condition as revealed with the Tukey post-hoc test, with P < 0.05, P < 0.01, and P < 0.001, respectively*.

In both the young adults and the elderly, statistical analysis showed that there was no significant main effect of the group or treatment nor significant interaction between these two factors on the execution phase duration, step length, and progression velocity at foot contact.

### Stability

In both the young adults and the elderly, no baseline differences were found between the WBV and sham groups (*p* > 0.05) for all the variables (cf. Table 3).

In the elderly, statistical analysis showed that there was no significant effect of the group or treatment nor any significant interaction between these two factors on the MOS and related components (i.e., step width, mediolateral COM position, and velocity at foot-contact). In contrast, in the young adults, there was a significant effect of the treatment, along with a significant group X treatment interaction on the following variables: MOS [*F*_(1, 39)_ = 5.14, *p* = 0.03; *F*_(1, 39)_ = 5.62, *p* = 0.02, respectively], mediolateral COM position [*F*_(1, 39)_ = 7.32, *p* = 0.01, *F*_(1, 39)_ = 5.19, *p* = 0.03, respectively], and mediolateral COM velocity at foot-contact [*F*_(1, 39)_ = 10.14, *p* < 0.01, *F*_(1, 39)_ = 4.51, *p* = 0.04; respectively]. *Post-hoc* analysis further revealed that these interactions could be ascribed to the result that none of these variables were significantly different between the pre and the post-treatment condition for the sham group, while they were statistically different for the WBV group. Specifically, the mediolateral COM position (*p* < 0.01) and the mediolateral COM velocity at foot-contact (*p* < 0.01) were both significantly smaller in the post-treatment condition than in the pre-treatment condition for the WBV group, while the MOS was significantly larger (*p* < 0.05; cf. Table 3).

### Stance Leg Stiffness

In both the young adults and the elderly, no baseline difference was found between the WBV and sham groups (*p* > 0.05; cf. Table 3). In the elderly, statistical analysis showed that there was no significant effect of the group or treatment nor any significant interaction between these two factors on the leg stiffness. In contrast, in the young adults, there was a significant effect of the treatment [*F*_(1, 39)_ = 4.15, *p* < 0.05) along with a significant group X treatment interaction [*F*_(1, 39)_ = 4.19, *p* = 0.04] on this variable. *Post-hoc* analysis further revealed that this interaction could be ascribed to the result that leg stiffness was not significantly different between the pre and the post-treatment condition for the sham group, while it was statistically larger for the WBV group (*p* < 0.05). In addition, results showed that, in the WBV group post-treatment condition, the stance leg stiffness in young adults was significantly correlated with the amplitude of mediolateral APA (*r* = 0.61, *p* < 0.01), i.e., the larger the leg stiffness, the larger the COP shift (Figure 2).

## Discussion

In the paragraph that follows, the coherence of biomechanical data and their implication in terms of advanced postural control knowledge in young adults and elderly are discussed.

### Effect of Acute WBV Application on APA in Young Adults

In young adults, results showed that the amplitude of the mediolateral APA, expressed in terms of peak COP shift, was increased in the WBV group post-treatment. The difference between the pre- and the post-treatment condition reached up to 16.1%, which may be considered as clinically significant. The result that no difference was observed between the pre-treatment and the post-treatment condition in the sham group discards a placebo or a practice effect due to the repetition of the task. The increased mediolateral APA could neither be ascribed to an increase in the progression velocity (27) or to an increase in the duration of the execution phase of gait initiation (26, 46, 47, 72). Results indeed showed that there was no significant effect of the WBV application on these two parameters. In contrast to the mediolateral APA amplitude, the mediolateral APA duration did not change in the WBV group post-treatment. Participants were therefore able to generate larger APA within the same duration, i.e., the APA efficacy was increased post-treatment. In line with this statement, results showed that the mediolateral COM velocity at the time of swing foot-off, which is known to depend on the spatiotemporal parameters of APA (27, 72), was larger following the WBV application. As this initial COM velocity is directed toward the stance leg, it acts to minimize the COM fall in the opposite direction (i.e., toward the swing leg) during the execution phase under the gravity effect (26, 49). The stabilizing function of APA was thus improved by acute WBV application.

The spatiotemporal parameters of APA are also known to be dependent on the somatosensory inputs arising from the lower limbs and trunk (52–55, 74–78). One can therefore wonder whether the positive effect on APA amplitude reported in this study could be ascribed to a change in the somatosensory inputs induced by the WBV application. While the current literature on healthy subjects repeatedly reported a detrimental effect of acute WBV application on the cutaneous lower limbs sensitivity (16, 79–81), it seems that acute WBV application does not influence lower limbs and trunk proprioception (17, 80–83); note that a detrimental effect on trunk proprioception was reported by Li et al. (84). Now, plantar sensitivity and limb proprioception were not investigated in the present study. However, an alteration of these inputs in the WBV group post-treatment (if any) may likely not be responsible for the changes in the mediolateral APA amplitude reported in the present study. For example, Lin and Yang (85) showed that the amplitude and the duration of mediolateral APA for gait initiation were both reduced (and not increased or unchanged, respectively, as in the present study) following plantar desensitization induced by cold water immersion. A decrease in the mediolateral APA for stepping was also reported when the ankle muscles acting in the mediolateral direction were vibrated to induce alteration of the proprioceptive afferent inflow from Ia fibers (53). In the present study, participants were exposed to WBV but, because of the standing posture with the feet on the vibrating force-plate, the ankle muscles were strongly submitted to the vibrations as in this latter study. The authors proposed that the proprioceptive information induced by vibration and afferent inflow related to body movement exaggerated sense of movement at the ankle. This biased perception led to a decrease in the APA spatiotemporal features. Similarly, a decrease in the postural response during balance recovery following cutaneous and muscular deafferentation experimentally induced by foot anesthesia and leg ischemia, respectively, was reported by Thoumie and Do (77). Finally, proprioceptive perturbation induced by experimental pain applied to the *tibialis anterior* (86) or acute fatigue of the same ankle muscles (78) were both shown to induce a decrease in the APA amplitude for gait initiation.

Globally, the above mentioned studies suggest that the increased mediolateral APA observed in the present research could probably not be ascribed to an (eventual) alteration of the somatosensory inputs induced by acute WBV application. Although not measured in these latter studies, it is possible that a major difference between these studies and the present one lays in the changes in the leg stiffness post-treatment. The stance leg stiffness was indeed increased by 16.8% during the execution phase of gait initiation following the WBV application, which may not be the case in the above mentioned studies due to the different treatments. In the following paragraph, it is argued that this change in the mechanical feature of the stance leg might be responsible for the increased mediolateral APA.

### Effect of Acute WBV Application on Stance Leg Stiffness and Relationship With APA and Stability in Young Adults

As for APA, the increased stance leg stiffness observed in the WBV group post-treatment could not be ascribed to a placebo or a practice effect since no change in this parameter could be detected in the sham group pre-treatment vs. post-treatment. This increased leg stiffness might possibly be related to a “TVR” induced by the activation of leg muscle spindles sensitive to vibrations (56, 57). The effect of WBV on limb stiffness is however controversial in the literature. In human, no effect of acute WBV on stiffness has been reported on patellar tendon (87), hamstring, quadriceps (88), and triceps surae (89) and in lower limbs during hoping (90). In contrast, WBV has been shown to increase area and stiffness of the flexor carpi ulnaris tendon in rats (91) and in the Achille tendon of older women (8). Colson and Petit (90) concluded that further studies should be undertaken to ascertain the effectiveness of WBV on lower limbs stiffness. It is possible that the effect of acute WBV application on muscle stiffness depends on the task to be done and the muscle tested (and particularly its content in spindles), but also on the mechanical features of the vibrations (amplitude, frequency, duration).

Whatever its origin, previous modeling study showed that increasing stance leg stiffness has the potential to induce instability during gait initiation (26). The current results showed that acute WBV application induced two opposite effects on postural stabilization: a *stabilizing* effect via an increase in the mediolateral APA amplitude, and a *destabilizing* effect via an increase in stance leg stiffness. Results showed that the combined action of these two opposite effects resulted in an increased stability during gait initiation, as assessed with the larger mediolateral margin of stability post-treatment. This increase reached 21.4%, which can be considered as clinically relevant. This finding suggests that the negative effect of WBV application on leg stiffness and on related-stability was (over)compensated by the larger mediolateral APA. Furthermore, results showed that these two balance control parameters were positively correlated, i.e., the larger the stance leg stiffness, the larger the mediolateral APA amplitude. Based on these findings, the authors propose that the increased stance leg stiffness induced by the WBV application was taken into account in the programming of the mediolateral APA. In this hypothesis, WBV application would induce a postural constraint in the form of an increased stance leg stiffness requiring motor adaptation to maintain stability. This interpretation is in agreement with the hypothesis that balance control mechanisms are interdependent within the postural system, i.e., they may compensate each other in case one component (here the leg stiffness) is perturbed (31, 92–94). This interdependence would be necessary to maintain an optimal control of stability in situations where a constraint is applied to the postural system. This statement is supported by recent studies. For example, during stepping over an obstacle in reaction to sudden force-plate translation, Zettel et al. (46, 47) reported that the drastic reduction of the mediolateral APA amplitude due to the urgency to clear the swing foot from the support surface did not result in a greater instability because it was compensated by larger step width, which corresponds to another form of balance control mechanism. In the same vein, Artico et al. (94) reported that the amplitude of mediolateral APA for gait initiation with the goal to clear an obstacle depended on whether participants had to strike the ground with a “rear foot strategy” (where the swing heel strikes first, RFS) or a “forefoot strategy” (where the toe strikes first, FFS). More specifically, it was found that the amplitude of the mediolateral APA was lower in the FFS than in the RFS condition. Striking the support surface with the toe first mechanically enlarged by a few centimeters the mediolateral base of support compared to striking with the heel first, thus making the control of stability in the final posture easier. As a consequence, the need to develop large mediolateral APA was lessened in the FFS condition as compared to the RFS condition to maintain stability. Because APA has an energy cost (46, 47, 95, 96), it was proposed that the CNS would reduce them in the former condition for economical purpose. Similar statement was proposed to explain why APA for upper limb task are depressed when the postural stability is made higher by an additional thoracic support (97). The mediolateral APA would be programmed according to the swing foot strike strategy and related-stability, thus revealing an interdependence between these two mechanisms. Globally, the current results thus add to the growing evidence that dynamic stability during gait initiation may share a principle of *homeostatic*-like regulation similar to most physiological variables, where separate mechanisms need to be coordinated to ensure stabilization of vital variables, here postural stability (31).

### Effects of Acute WBV on Postural Organization of Gait Initiation in the Elderly

In contrast to young adults, no significant effects of acute WBV application for any of the biomechanical variables recorded was observed in the elderly. This finding may a priori contrast with current literature that instead tends to show a beneficial effect of WBV application on postural control in static position and on various clinical locomotor tests [for review (98)]. Now, it is noteworthy that these latter studies used long-term WBV treatments and not single bout as in the present study. It is therefore not excluded that long-term WBV application would result in dynamic stability improvement in the elderly. The result that no increase in stance leg stiffness was observed in the WBV group post-treatment may possibly be ascribed to the progressive alteration with aging in the central structures (e.g., spinal motoneurons) (99, 100), somatosensory receptors of the lower limbs (e.g., spindles and mechanoreceptors of the plantar foot sole) (101, 102), and/or sensory-motor pathways involved in the TVR reflex (103). This alteration would make elderly's stance leg stiffness less sensitive to acute WBV application than young adults. As a consequence, no motor adaptation e.g., in the form of larger APA or larger step width would be required to maintain stability since it was no further challenged by the WBV application. The current results however suggest that studies focusing on the effects of long-term WBV application in elderly should take into consideration the possibility that leg stiffness may be increased by this treatment. In case participants are not able to develop motor adaptation, instability may occur during locomotor tasks with increasing risks of falls.

## Conclusion

The present results showed that the effects of acute WBV application on the postural organization of gait initiation depend on the age of participants. In young healthy adults, a positive effect was observed on dynamic stability, while no effect was observed in the elderly. The positive effect was ascribed to an increase in the mediolateral APA following WBV application which overcompensated the potentially destabilizing effect of the increased stance leg stiffness. WBV application may thus be efficient to improve dynamic stability but at the condition that participants are able to develop adaptive changes in balance control mechanisms. In elderly, no anticipatory (nor corrective) postural adaptation was required since acute WBV application did not elicit change in the stance leg stiffness. Globally, the present finding is therefore in agreement with the hypothesis that balance control mechanisms are interdependent within the postural system, i.e., they may compensate each other in case one component (here the leg stiffness) is perturbed.

## Data Availability Statement

The datasets generated for this study are available on request to the corresponding author.

## Ethics Statement

The studies involving human participants were reviewed and approved by CIAMS Research Unit, Equipe d'Accueil (EA) 4532. The patients/participants provided their written informed consent to participate in this study. Written informed consent was obtained from the individual(s) for the publication of any potentially identifiable images or data included in this article.

## Author Contributions

AD, TV, MF, GL, LC, RA, FG, PF, and EY contributed with project creation, data collection, data analysis, drafted the manuscript, discussed the results, and participated in the revision of the manuscript.

### Conflict of Interest

The authors declare that the research was conducted in the absence of any commercial or financial relationships that could be construed as a potential conflict of interest.

## References

[1] Mahieu NN Witvrouw E Van de Voorde D Michilsens D Arbyn V Van den Broecke W. Improving strength and postural control in young skiers: whole-body vibration versus equivalent resistance training. J Athl Train. (2006) 41:286–93. 17043697 PMC1569559

[2] Melnyk M Kofler B Faist M Hodapp M Gollhofer A. Effect of a whole-body vibration session on knee stability. Int J Sports Med. (2008) 29:839–44. 10.1055/s-2008-1038405 18401809

[3] Ritzmann R Kramer A Bernhardt S Gollhofer A. Whole body vibration training–improving balance control and muscle endurance. PLoS ONE. (2014) 9:e89905. 10.1371/journal.pone.0089905 24587114 PMC3935964

[4] Ritzmann R Gollhofer A Kramer A. The influence of vibration type, frequency, body position and additional load on the neuromuscular activity during whole body vibration. Eur J Appl Physiol. (2013) 113:1–11. 10.1007/s00421-012-2402-0 22538279

[5] Torvinen S Kannus P Sievänen H Järvinen TAH Pasanen M Kontulainen S. Effect of four-month vertical whole body vibration on performance and balance. Med Sci Sports Exerc. (2002) 34:1523–8. 10.1097/00005768-200209000-00020 12218749

[6] Torvinen S Sievänen H Järvinen TA Pasanen M Kontulainen S Kannus P. Effect of 4-min vertical whole body vibration on muscle performance and body balance: a randomized cross-over study. Int J Sports Med. (2002) 23:374–9. 10.1055/s-2002-33148 12165890

[7] Torvinen S Kannus P Sievänen H Järvinen TA Pasanen M Kontulainen S. Effect of 8-month vertical whole body vibration on bone, muscle performance, and body balance: a randomized controlled study. J Bone Miner Res. (2003) 18:876–84. 10.1359/jbmr.2003.18.5.876 12733727

[8] Han S-W Lee D-Y Choi D-S Han B Kim J-S Lee H-D. Asynchronous alterations of muscle force and tendon stiffness following 8 weeks of resistance exercise with whole-body vibration in older women. J Aging Phys Act. (2017) 25:287–94. 10.1123/japa.2016-0149 27768508

[9] Rees SS Murphy AJ Watsford ML. Effects of whole body vibration on postural steadiness in an older population. J Sci Med Sport. (2009) 12:440–4. 10.1016/j.jsams.2008.02.002 18550436

[10] Rees SS Murphy AJ Watsford ML. Effects of whole-body vibration exercise on lower-extremity muscle strength and power in an older population: a randomized clinical trial. Phys Ther. (2008) 88:462–70. 10.2522/ptj.20070027 18218826

[11] Roelants M Delecluse C Verschueren SM. Whole-body-vibration training increases knee-extension strength and speed of movement in older women. J Am Geriatr Soc. (2004) 52:901–8. 10.1111/j.1532-5415.2004.52256.x 15161453

[12] Rogan S Radlinger L Hilfiker R Schmidtbleicher D de Bie RA de Bruin ED. Feasibility and effects of applying stochastic resonance whole-body vibration on untrained elderly: a randomized crossover pilot study. BMC Geriatr. (2015) 15:25. 10.1186/s12877-015-0021-4 25886789 PMC4371632

[13] Yang F King GA Dillon L Su X. Controlled whole-body vibration training reduces risk of falls among community-dwelling older adults. J Biomech. (2015) 48:3206–12. 10.1016/j.jbiomech.2015.06.029 26189095

[14] Rogan S Hilfiker R Schenk A Vogler A Taeymans J. Effects of whole-body vibration with stochastic resonance on balance in persons with balance disability and falls history - a systematic review. Res Sports Med. (2014) 22:294–313. 10.1080/15438627.2014.919504 24950116

[15] Piecha M Juras G Król P Sobota G Polak A Bacik B. The effect of a short-term and long-term whole-body vibration in healthy men upon the postural stability. PLoS ONE. (2014) 9:e88295. 10.1371/journal.pone.0088295 24520362 PMC3919744

[16] Schlee G Reckmann D Milani TL. Whole body vibration training reduces plantar foot sensitivity but improves balance control of healthy subjects. Neurosci Lett. (2012) 506:70–3. 10.1016/j.neulet.2011.10.051 22061837

[17] Hiroshige K Mahbub MH Harada N. Effects of whole-body vibration on postural balance and proprioception in healthy young and elderly subjects: a randomized cross-over study. J Sports Med Phys Fitness. (2014) 54:216–24. 24509994

[18] Orr R. The effect of whole body vibration exposure on balance and functional mobility in older adults: a systematic review and meta-analysis. Maturitas. (2015) 80:342–58. 10.1016/j.maturitas.2014.12.020 25631348

[19] Rogan S Taeymans J Radlinger L Naepflin S Ruppen S Bruelhart Y Hilfiker R. Effects of whole-body vibration on postural control in elderly: an update of a systematic review and meta-analysis. Arch Gerontol Geriatr. (2017) 73:95–112. 10.1016/j.archger.2017.07.022 28800481

[20] Rogan S Hilfiker R Herren K Radlinger L de Bruin ED. Effects of whole-body vibration on postural control in elderly: a systematic review and meta-analysis. BMC Geriatr. (2011) 11:72. 10.1186/1471-2318-11-72 22054046 PMC3229447

[21] Blum L Korner-Bitensky N. Usefulness of the Berg Balance Scale in stroke rehabilitation: a systematic review. Phys Ther. (2008) 88:559–66. 10.2522/ptj.20070205 18292215

[22] Mancini M Horak FB. The relevance of clinical balance assessment tools to differentiate balance deficits. Eur J Phys Rehabil Med. (2010) 46:239–48. 20485226 PMC3033730

[23] Brenière Y Cuong Do M Bouisset S. Are dynamic phenomena prior to stepping essential to walking? J Mot Behav. (1987) 19:62–76. 10.1080/00222895.1987.10735400 23944913

[24] Breniere Y Do MC. When and how does steady state gait movement induced from upright posture begin? J Biomech. (1986) 19:1035–40. 10.1016/0021-9290(86)90120-X 3818673

[25] Lepers R Brenière Y. The role of anticipatory postural adjustments and gravity in gait initiation. Exp Brain Res. (1995) 107:118–24. 10.1007/BF00228023 8751069

[26] Yiou E Artico R Teyssedre CA Labaune O Fourcade P. Anticipatory postural control of stability during gait initiation over obstacles of different height and distance made under reaction-time and self-initiated instructions. Front Hum Neurosci. (2016) 10:449. 10.3389/fnhum.2016.00449 27656138 PMC5013047

[27] Caderby T Yiou E Peyrot N Begon M Dalleau G. Influence of gait speed on the control of mediolateral dynamic stability during gait initiation. J Biomech. (2014) 47:417–23. 10.1016/j.jbiomech.2013.11.011 24290175

[28] Chong RKY Chastan N Welter M-L Do M-C. Age-related changes in the center of mass velocity control during walking. Neurosci Lett. (2009) 458:23–7. 10.1016/j.neulet.2009.04.022 19442871

[29] Honeine J-L Schieppati M Gagey O Do M-C. The functional role of the triceps surae muscle during human locomotion. PLoS ONE. (2013) 8:52943. 10.1371/journal.pone.0052943 23341916 PMC3547017

[30] Honeine J-L Schieppati M Gagey O Do M-C. By counteracting gravity, triceps surae sets both kinematics and kinetics of gait. Physiol Rep. (2014) 2:e00229. 10.1002/phy2.229 24744898 PMC3966244

[31] Yiou E Caderby T Delafontaine A Fourcade P Honeine J-L. Balance control during gait initiation: state-of-the-art and research perspectives. World J Orthop. (2017) 8:815–28. 10.5312/wjo.v8.i11.815 29184756 PMC5696609

[32] Barry E Galvin R Keogh C Horgan F Fahey T. Is the Timed Up and Go test a useful predictor of risk of falls in community dwelling older adults: a systematic review and meta-analysis. BMC Geriatr. (2014) 14:14. 10.1186/1471-2318-14-14 24484314 PMC3924230

[33] Topper AK Maki BE Holliday PJ Are activity-based assessments of balance and gait in the elderly predictive of risk of falling and/or type of fall? J Am Geriatr Soc. (1993) 41:479–87. 10.1111/j.1532-5415.1993.tb01881.x 8486878

[34] Delafontaine A Gagey O Colnaghi S Do M-C Honeine J-L. Rigid ankle foot orthosis deteriorates mediolateral balance control and vertical braking during gait initiation. Front Hum Neurosci. (2017) 11:214. 10.3389/fnhum.2017.00214 28503144 PMC5408009

[35] Honeine J-L Schieppati M Crisafulli O Do M-C. The neuro-mechanical processes that underlie goal-directed medio-lateral APA during gait initiation. Front Hum Neurosci. (2016) 10:445. 10.3389/fnhum.2016.00445 27642280 PMC5015477

[36] Cau N Cimolin V Galli M Precilios H Tacchini E Santovito C. Center of pressure displacements during gait initiation in individuals with obesity. J NeuroEng Rehabil. (2014) 11:82. 10.1186/1743-0003-11-82 24885764 PMC4026057

[37] Zago M Capodaglio P Ferrario C Tarabini M Galli M. Whole-body vibration training in obese subjects: a systematic review. PLoS ONE. (2018) 13:e0202866. 10.1371/journal.pone.0202866 30183742 PMC6124767

[38] Cimolin V Cau N Galli M Santovito C Grugni G Capodaglio P. Gait initiation and termination strategies in patients with Prader-Willi syndrome. J Neuroeng Rehabil. (2017) 14:44. 10.1186/s12984-017-0257-7 28535762 PMC5442593

[39] Sousa Silva A Santos R. Reliability of two methods for identifying the postural phase of gait initiation in healthy and poststroke subjects. J Appl Biomech. (2015) 31:349–56. 10.1123/jab.2014-0222 26033346

[40] Corsi C Cimolin V Capodaglio P Condoluci C Galli M. A biomechanical study of gait initiation in Down syndrome. BMC Neurol. (2019) 19:66. 10.1186/s12883-019-1288-4 30987596 PMC6466789

[41] Chang H Krebs DE. Dynamic balance control in elders: gait initiation assessment as a screening tool. Arch Phys Med Rehabil. (1999) 80:490–4. 10.1016/S0003-9993(99)90187-9 10326909

[42] Hass CJ Waddell DE Wolf SL Juncos JL Gregor RJ. Gait initiation in older adults with postural instability. Clin Biomech. (2008) 23:743–53. 10.1016/j.clinbiomech.2008.02.012 18407387 PMC2954654

[43] Lee J Webb G Shortland AP Edwards R Wilce C Jones GD Reliability and feasibility of gait initiation centre-of-pressure excursions using a Wii® Balance Board in older adults at risk of falling. Aging Clin Exp Res. (2019) 31:257–63. 10.1007/s40520-018-0945-6 29667154 PMC6373388

[44] Mbourou GA Lajoie Y Teasdale N. Step length variability at gait initiation in elderly fallers and non-fallers, and young adults. Gerontology. (2003) 49:21–6. 10.1159/000066506 12457046

[45] Crenna P Frigo C. A motor programme for the initiation of forward-oriented movements in humans. J Physiol. (1991) 437:635–53. 10.1113/jphysiol.1991.sp018616 1890653 PMC1180068

[46] Zettel JL McIlroy WE Maki BE. Environmental constraints on foot trajectory reveal the capacity for modulation of anticipatory postural adjustments during rapid triggered stepping reactions. Exp Brain Res. (2002) 146:38–47. 10.1007/s00221-002-1150-5 12192576

[47] Zettel JL McIlroy WE Maki BE. Can stabilizing features of rapid triggered stepping reactions be modulated to meet environmental constraints? Exp Brain Res. (2002) 145:297–308. 10.1007/s00221-002-1083-z 12136379

[48] Lyon IN Day BL. Predictive control of body mass trajectory in a two-step sequence. Exp Brain Res. (2005) 161:193–200. 10.1007/s00221-004-2058-z 15480597

[49] Lyon IN Day BL. Control of frontal plane body motion in human stepping. Exp Brain Res. (1997) 115:345–56. 10.1007/PL00005703 9224862

[50] Tisserand R Robert T Chabaud P Bonnefoy M Chèze L. Elderly fallers enhance dynamic stability through anticipatory postural adjustments during a choice stepping reaction time. Front Hum Neurosci. (2016) 10:613. 10.3389/fnhum.2016.00613 27965561 PMC5126045

[51] Yiou E Caderby T Hussein T. Adaptability of anticipatory postural adjustments associated with voluntary movement. World J Orthop. (2012) 3:75–86. 10.5312/wjo.v3.i6.75 22720267 PMC3377909

[52] Roden-Reynolds DC Walker MH Wasserman CR Dean JC. Hip proprioceptive feedback influences the control of mediolateral stability during human walking. J Neurophysiol. (2015) 114:2220–9. 10.1152/jn.00551.2015 26289467 PMC4600962

[53] Ruget H Blouin J Coyle T Mouchnino L. Modulation of proprioceptive inflow when initiating a step influences postural adjustments. Exp Brain Res. (2010) 201:297–305. 10.1007/s00221-009-2035-7 19834696

[54] Ruget H Blouin J Teasdale N Mouchnino L. Can prepared anticipatory postural adjustments be updated by proprioception? Neuroscience. (2008) 155:640–8. 10.1016/j.neuroscience.2008.06.021 18620030

[55] Saradjian AH Tremblay L Perrier J Blouin J Mouchnino L. Cortical facilitation of proprioceptive inputs related to gravitational balance constraints during step preparation. J Neurophysiol. (2013) 110:397–407. 10.1152/jn.00905.2012 23576699

[56] Burke D Hagbarth KE Löfstedt L Wallin BG. The responses of human muscle spindle endings to vibration during isometric contraction. J Physiol. (1976) 261:695–711. 10.1113/jphysiol.1976.sp011581 135841 PMC1309167

[57] Hagbarth KE Eklund G. Tonic vibration reflexes (TVR) in spasticity. Brain Res. (1966) 2:201–3. 10.1016/0006-8993(66)90029-1 5968925

[58] Fowler BD Palombo KTM Feland JB Blotter JD. Effects of whole-body vibration on flexibility and stiffness: a literature review. Int J Exerc Sci. (2019) 12:735–47. 31156749 10.70252/TJVC4921 PMC6533098

[59] Roschel H Barroso R Tricoli V Batista MAB Acquesta FM Serrão JC Ugrinowitsch C. Effects of strength training associated with whole-body vibration training on running economy and vertical stiffness. J Strength Cond Res. (2015) 29:2215–20. 10.1519/JSC.0000000000000857 25627640

[60] Wang P Yang X Yang Y Yang L Zhou Y Liu C. Effects of whole body vibration on pain, stiffness and physical functions in patients with knee osteoarthritis: a systematic review and meta-analysis. Clin Rehabil. (2015) 29:939–51. 10.1177/0269215514564895 25525066

[61] Ness LL Field-Fote EC. Whole-body vibration improves walking function in individuals with spinal cord injury: a pilot study. Gait Posture. (2009) 30:436–40. 10.1016/j.gaitpost.2009.06.016 19648013 PMC2753701

[62] van Nes IJW Latour H Schils F Meijer R van Kuijk A Geurts ACH. Long-term effects of 6-week whole-body vibration on balance recovery and activities of daily living in the postacute phase of stroke: a randomized, controlled trial. Stroke. (2006) 37:2331–5. 10.1161/01.STR.0000236494.62957.f3 16902175

[63] van Nes IJW Geurts ACH Hendricks HT Duysens J. Short-term effects of whole-body vibration on postural control in unilateral chronic stroke patients: preliminary evidence. Am J Phys Med Rehabil. (2004) 83:867–73. 10.1097/01.PHM.0000140801.23135.09 15502741

[64] Farley CT Morgenroth DC. Leg stiffness primarily depends on ankle stiffness during human hopping. J Biomech. (1999) 32:267–73. 10.1016/S0021-9290(98)00170-5 10093026

[65] Morasso PG Schieppati M. Can muscle stiffness alone stabilize upright standing? J Neurophysiol. (1999) 82:1622–6. 10.1152/jn.1999.82.3.1622 10482776

[66] Brenière Y Do MC. Control of gait initiation. J Mot Behav. (1991) 23:235–40. 10.1080/00222895.1991.9942034 14766505

[67] Caderby T Yiou E Peyrot N Bonazzi B Dalleau G. Detection of swing heel-off event in gait initiation using force-plate data. Gait Posture. (2013) 37:463–6. 10.1016/j.gaitpost.2012.08.011 22980912

[68] Delafontaine A Honeine J-L Do M-C Gagey O Chong RK. Comparative gait initiation kinematics between simulated unilateral and bilateral ankle hypomobility: does bilateral constraint improve speed performance? Neurosci Lett. (2015) 603:55–9. 10.1016/j.neulet.2015.07.016 26197055

[69] Yiou E Teyssèdre C Artico R Fourcade P. Comparison of base of support size during gait initiation using force-plate and motion-capture system: a Bland and Altman analysis. J Biomech. (2016) 49:4168–72. 10.1016/j.jbiomech.2016.11.008 27855983

[70] Hof AL Gazendam MGJ Sinke WE. The condition for dynamic stability. J Biomech. (2005) 38:1–8. 10.1016/j.jbiomech.2004.03.025 15519333

[71] Caderby T Yiou E Peyrot N de Viviés X Bonazzi B Dalleau G. Effects of changing body weight distribution on mediolateral stability control during gait initiation. Front Hum Neurosci. (2017) 11:127. 10.3389/fnhum.2017.00127 28396629 PMC5366317

[72] Yiou E Fourcade P Artico R Caderby T Influence of temporal pressure constraint on the biomechanical organization of gait initiation made with or without an obstacle to clear. Exp Brain Res. (2016) 234:1363–75. 10.1007/s00221-015-4319-4 25990822

[73] Winter DA Patla AE Frank JS. Assessment of balance control in humans. Med Prog Technol. (1990) 16:31–51. 2138696

[74] Ditcharles S Yiou E Delafontaine A Hamaoui A. Short-term effects of thoracic spine manipulation on the biomechanical organisation of gait initiation: a randomized pilot study. Front Hum Neurosci. (2017) 11:343. 10.3389/fnhum.2017.00343 28713254 PMC5491951

[75] Do MC Bussel B Breniere Y. Influence of plantar cutaneous afferents on early compensatory reactions to forward fall. Exp Brain Res. (1990) 79:319–24. 10.1007/BF00608241 2323379

[76] Mouchnino L Blouin J. When standing on a moving support, cutaneous inputs provide sufficient information to plan the anticipatory postural adjustments for gait initiation. PLoS ONE. (2013) 8:e55081. 10.1371/journal.pone.0055081 23390513 PMC3563658

[77] Thoumie P Do MC. Changes in motor activity and biomechanics during balance recovery following cutaneous and muscular deafferentation. Exp Brain Res. (1996) 110:289–97. 10.1007/BF00228559 8836692

[78] Yiou E Ditcharles S Le Bozec S. Biomechanical reorganisation of stepping initiation during acute dorsiflexor fatigue. J Electromyogr Kinesiol. (2011) 21:727–33. 10.1016/j.jelekin.2011.04.008 21605984

[79] Alfuth M Beiring A Klein D Rosenbaum D Acute effects of whole body vibration on foot sole sensitivity and plantar pressures during gait initiation. J Foot Ankle Res. (2012) 5:O24 10.1186/1757-1146-5-S1-O24

[80] Pollock RD Provan S Martin FC Newham DJ. The effects of whole body vibration on balance, joint position sense and cutaneous sensation. Eur J Appl Physiol. (2011) 111:3069–77. 10.1007/s00421-011-1943-y 21455611

[81] Pollock RD Provan S Martin FC Newham DJ Whole body vibration; short-term effects on balance, proprioception and cutaneous sensation in healthy young adults. Proc of The Phys Soc. (2010) 19:3069–77. 10.3138/ptc.2014-77

[82] Hannah R Minshull C Folland JP Whole-body vibration does not influence knee joint neuromuscular function or proprioception. Scand J Med Sci Sports. (2013) 23:96–104. 10.1111/j.1600-0838.2011.01361.x 21819446

[83] Lee TY Chow DHK. Effects of whole body vibration on spinal proprioception in normal individuals. Conf Proc IEEE Eng Med Biol Soc. (2013) 2013:4989–92. 10.1109/EMBC.2013.6610668 24110855

[84] Li L Lamis F Wilson SE Whole-body vibration alters proprioception in the trunk. Int J Ind Ergon. (2008) 38:792–800. 10.1016/j.ergon.2007.10.010

[85] Lin SI Yang WC. Effect of plantar desensitization on postural adjustments prior to step initiation. Gait Posture. (2011) 34:451–6. 10.1016/j.gaitpost.2011.06.016 21795046

[86] Madeleine P Voigt M Arendt-Nielsen L. Reorganisation of human step initiation during acute experimental muscle pain. Gait Posture. (1999) 10:240–7. 10.1016/S0966-6362(99)00036-3 10567756

[87] Rieder F Wiesinger H-P Kösters A Müller E Seynnes OR. Immediate effects of whole body vibration on patellar tendon properties and knee extension torque. Eur J Appl Physiol. (2016) 116:553–61. 10.1007/s00421-015-3316-4 26708361

[88] Siu PM Tam BT Chow DH Guo J-Y Huang Y-P Zheng Y-P Wong SH. Immediate effects of 2 different whole-body vibration frequencies on muscle peak torque and stiffness. Arch Phys Med Rehabil. (2010) 91:1608–15. 10.1016/j.apmr.2010.07.214 20875522

[89] Cronin JB Oliver M McNair PJ Muscle stiffness and injury effects of whole body vibration. Phys Ther Sport. (2004) 5:68–74. 10.1016/S1466-853X(04)00020-3

[90] Colson SS Petit P-D. Lower limbs power and stiffness after whole-body vibration. Int J Sports Med. (2013) 34:318–23. 10.1055/s-0032-1311596 23143701

[91] Sandhu E Miles JD Dahners LE Keller BV Weinhold PS. Whole body vibration increases area and stiffness of the flexor carpi ulnaris tendon in the rat. J Biomech. (2011) 44:1189–91. 10.1016/j.jbiomech.2011.02.017 21396647

[92] Delafontaine A Fourcade P Honeine JL Ditcharles S Yiou E. Postural adaptations to unilateral knee joint hypomobility induced by orthosis wear during gait initiation. Sci Rep. (2018) 8:830. 10.1038/s41598-018-19151-1 29339773 PMC5770397

[93] Reimann H Fettrow TD Thompson ED Agada P McFadyen BJ Jeka JJ. Complementary mechanisms for upright balance during walking. PLoS ONE. (2017) 12:e0172215. 10.1371/journal.pone.0172215 28234936 PMC5325219

[94] Artico R Fourcade P Teyssedre CA Delafontaine A Yiou E Influence of swing foot strike pattern on balance control mechanisms during gait initiation over an obstacle to clear. In: Progress in Motor Control. Amsterdam (2019) Available online at: https://10times.com/pmc-netherlands

[95] Aruin AS Forrest WR Latash ML. Anticipatory postural adjustments in conditions of postural instability. Electroencephalogr Clin Neurophysiol. (1998) 109:350–9. 10.1016/S0924-980X(98)00029-0 9751298

[96] Nouillot P Bouisset S Do MC. Do fast voluntary movements necessitate anticipatory postural adjustments even if equilibrium is unstable? Neurosci Lett. (1992) 147:1–4. 10.1016/0304-3940(92)90760-5 1480314

[97] Cordo PJ Nashner LM. Properties of postural adjustments associated with rapid arm movements. J Neurophysiol. (1982) 47:287–302. 10.1152/jn.1982.47.2.287 7062101

[98] Fischer M Vialleron T Laffaye G Fourcade P Hussein T Chèze L. Long-term effects of whole-body vibration on human gait: a systematic review and meta-analysis. Front Neurol. (2019) 10:627. 10.3389/fneur.2019.00627 31316447 PMC6611385

[99] Varma VR Hausdorff JM Studenski SA Rosano C Camicioli R Alexander NB. Aging, the central nervous system, and mobility in older adults: interventions. J Gerontol A Biol Sci Med Sci. (2016) 71:1451–8. 10.1093/gerona/glw080 27154905 PMC5055648

[100] Edström E Altun M Bergman E Johnson H Kullberg S Ramírez-León V. Factors contributing to neuromuscular impairment and sarcopenia during aging. Physiol Behav. (2007) 92:129–35. 10.1016/j.physbeh.2007.05.040 17585972

[101] Liu J-X Eriksson P-O Thornell L-E Pedrosa-Domellöf F. Fiber content and myosin heavy chain composition of muscle spindles in aged human biceps brachii. J Histochem Cytochem. (2005) 53:445–54. 10.1369/jhc.4A6257.2005 15805419

[102] Peters RM McKeown MD Carpenter MG Inglis JT. Losing touch: age-related changes in plantar skin sensitivity, lower limb cutaneous reflex strength, and postural stability in older adults. J Neurophysiol. (2016) 116:1848–58. 10.1152/jn.00339.2016 27489366 PMC5144713

[103] Shaffer SW Harrison AL. Aging of the somatosensory system: a translational perspective. Phys Ther. (2007) 87:193–207. 10.2522/ptj.20060083 17244695

